# 2079. Maternal hybrid immunity to SARS-CoV-2 during pregnancy provides more durable infant antibody responses compared to natural infection alone

**DOI:** 10.1093/ofid/ofad500.149

**Published:** 2023-11-27

**Authors:** Sylvia M LaCourse, Erica A Wetzler, Morgan C Aurelio, Jaclyn N Escudero, Stacy Selke, Alex L Greninger, Erin Goecker, Sarina R Barnes, Isabel S Arnould, Ailyn C Pérez-Osorio, Alisa B Kachikis, Janet A Englund, Alison L Drake

**Affiliations:** University of Washington, Seattle, WA; University of Washington, Seattle, WA; University of Washington, Seattle, WA; University of Washington, Seattle, WA; University of Washington, Seattle, WA; UW Medicine, Seattle, WA; University of Washington, Seattle, WA; University of Washington, Seattle, WA; University of Washington, Seattle, WA; University of Washington, Seattle, WA; University of Washington Department of Obstetrics & Gynecology, Seattle, Washington; Seattle Children’s Hospital, Seattle, Washington; University of Washington, Seattle, WA

## Abstract

**Background:**

Immunity from natural infection and vaccination (hybrid) may provide more durable SARS-CoV-2 antibody responses; whether this increases durability of maternally-derived antibody responses in infants is unknown.

**Methods:**

Participants with prior SARS-CoV-2 infection in pregnancy (anti-nucleocapsid [anti-N] IgG+ on enrollment or prior RT-PCR+ or antigen+) were enrolled between January 2021-August 2022. Blood samples collected in pregnancy, delivery/birth, 0-< 3 and 3-6 months postpartum were tested for anti-S+ IgG by Abbott Architect (positive: ≥50 AU/mL) and neutralizing antibodies (serum dilution inhibited infection by 50% [ND50 heat] ≥20 and R^2^ ≥0.9).

**Results:**

Among 107 participants at enrollment in pregnancy, median age was 32 years (IQR 30-35) and median gestational age was 31 weeks (IQR 19.1–37.9). At delivery (median 19.7 weeks [IQR 14.3-30.0] from SARS CoV-2 diagnosis), unvaccinated participants and their infants were less likely to have anti-S IgG+ (maternal 87%; cord 86%) or neutralizing antibodies (maternal 86%; cord 75%) than vaccinated (≥1 dose) participants and their infants (maternal and cord both 100% anti-S IgG+ and neutralizing antibodies) (all p≤0.01). By 6 months of age, the proportion of infants of mothers who remained unvaccinated at birth with anti-S IgG+ and neutralizing antibody declined to 50% and 14% (anti-S IgG+ and neutralizing antibody, respectively) compared to 100% (anti-S IgG+ and neutralizing antibody) among infants with vaccinated mothers (all p< 0.01).

Infants with anti-S IgG+ or neutralizing antibodies born to unvaccinated mothers had lower median antibody levels at birth (anti-S IgG log_10_ 2.95 vs. 4.40 AU/ml; neutralization log_10_ 1:2.37 vs. 1:4.00, all p< 0.01) and through 6 months of age (anti-S IgG log_10_ 1.95 vs. 3.84 AU/ml, p< 0.01; log_10_ neutralization 1:1.34 vs. 1:3.20, p=0.11) vs. infants with mothers vaccinated prior to delivery.

Neutralizing antibody responses among A) pregnant people with prior SARS-CoV-2 infection during pregnancy and B) their infants by maternal vaccination status *†

**Figure:**
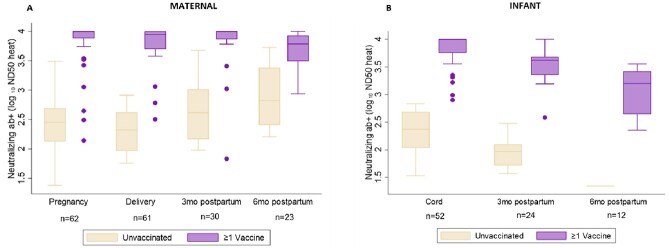

*Among participants and their infants with positive neutralizing antibodies (ND50 heat ≥20 and R2 ≥0.9). †Maternal vaccination status is at time of blood sample collection; for infants refers to maternal vaccination status at time of delivery.

**Conclusion:**

SARS-CoV-2 infection alone during pregnancy did not provide persistent antibody responses in infants through 6 months of age. Maternal vaccination provided more durable antibody responses in participants with prior SARS-CoV-2, potentially providing protection to vulnerable infants in the months prior to their own COVID-19 vaccine eligibility.

**Disclosures:**

**Sylvia M. LaCourse, MD, MPH**, Merck: Grant/Research Support **Alex L. Greninger, MD, PhD, MS, MPhil**, Abbott Diagnostics: central testing|Cepheid: central testing|Hologic Inc: central testing|Janssen Infectious Disease: central testing|Novavax, Inc.: central testing|Pfizer, Inc.: central testing **Alisa B. Kachikis, MD, MSc**, Merck: Grant/Research Support|Pfizer: Grant/Research Support **Janet A. Englund, MD**, Ark Biopharma: Advisor/Consultant|AstraZeneca: Advisor/Consultant|AstraZeneca: Grant/Research Support|GlaxoSmithKline: Grant/Research Support|Meissa Vaccines: Advisor/Consultant|Merck: Grant/Research Support|Moderna: Advisor/Consultant|Moderna: Grant/Research Support|Pfizer: Advisor/Consultant|Pfizer: Grant/Research Support|Sanofi Pasteur: Advisor/Consultant **Alison L. Drake, PhD, MPH**, Merck: Grant/Research Support

